# Mid‐life and late‐life vascular risk factor burden and neuropathology in old age

**DOI:** 10.1002/acn3.50936

**Published:** 2019-11-05

**Authors:** Sarah C. Conner, Matthew P. Pase, Herman Carneiro, Mekala R. Raman, Ann C. McKee, Victor E. Alvarez, Jamie M. Walker, Claudia L. Satizabal, Jayandra J. Himali, Thor D. Stein, Alexa Beiser, Sudha Seshadri

**Affiliations:** ^1^ Framingham Heart Study Framingham Massachusetts; ^2^ Department of Biostatistics Boston University School of Public Health Boston Massachusetts; ^3^ Melbourne Dementia Research Centre The Florey Institute for Neuroscience and Mental Health Melbourne Australia; ^4^ Centre for Human Psychopharmacology Swinburne University of Technology Melbourne Australia; ^5^ Faculty of Medicine Dentistry and Health Sciences The University of Melbourne Melbourne Australia; ^6^ Department of Medicine Boston University School of Medicine Boston Massachusetts; ^7^ Department of Neurology Boston University School of Medicine Boston Massachusetts; ^8^ Boston University Alzheimer's Disease and CTE Center Boston University School of Medicine Boston Massachusetts; ^9^ Department of Veterans Affairs Medical Center Bedford Massachusetts; ^10^ VA Boston Healthcare System Boston Massachusetts; ^11^ Department of Pathology and Laboratory Medicine Boston University School of Medicine Boston Massachusetts; ^12^ Glenn Biggs Institute for Alzheimer’s and Neurodegenerative Diseases UT Health San Antonio San Antonio Texas

## Abstract

**Objective:**

To determine whether vascular risk factor burden in mid‐ or late‐life associates with postmortem vascular and neurodegenerative pathologies in a community‐based sample.

**Methods:**

We studied participants from the Framingham Heart Study who participated in our voluntary brain bank program. Overall vascular risk factor burden was calculated using the Framingham Stroke Risk Profile (FSRP). Mid‐life FSRP was measured at 50 to 60 years of age. Following death, brains were autopsied and semi‐quantitatively assessed by board‐certified neuropathologists for cerebrovascular outcomes (cortical infarcts, subcortical infarcts, atherosclerosis, arteriosclerosis) and Alzheimer’s disease pathology (Braak stage, cerebral amyloid angiopathy, and neuritic plaque score). We estimated adjusted odds ratios between vascular risk burden (at mid‐life and before death) and neuropathological outcomes using logistic and proportional‐odds logistic models.

**Results:**

The median time interval between FSRP and death was 33.4 years for mid‐life FSRP and 4.4 years for final FSRP measurement before death. Higher mid‐life vascular risk burden was associated with increased odds of all cerebrovascular pathology, even with adjustment for vascular risk burden before death. Late‐life vascular risk burden was associated with increased odds of cortical infarcts (OR [95% CI]: 1.04 [1.00, 1.08]) and arteriosclerosis stage (OR [95% CI]: 1.03 [1.00, 1.05]). Mid‐life vascular risk burden was not associated with Alzheimer’s disease pathology, though late‐life vascular risk burden was associated with increased odds of higher Braak stage (OR [95% CI]: 1.03 [1.01, 1.05]).

**Interpretation:**

Mid‐life vascular risk burden was predictive of cerebrovascular but not Alzheimer’s disease neuropathology, even after adjustment for vascular risk factors before death.

## Introduction

Growing evidence points to mid‐life as a critical period by which vascular risk factors most strongly associate with brain health into old age. In the Framingham Heart Study (FHS), we recently showed that the association between higher vascular risk factor burden and lower brain volume was strongest when vascular risk factors were measured at younger ages.^1^ For example, vascular risk factors at age 45 strongly related to brain volume on MRI at age 85. Also in the FHS, mid‐life hypertension more strongly associates with late‐life dementia risk as compared to late‐life hypertension.^2^ Similarly, the Whitehall II study showed that high systolic blood pressure at age 50, but not 60 or 70, predicted a higher risk of late‐life dementia.^3^ However, these former studies focusing on clinical diagnosis or global brain volumes do not shed light on the underlying pathology. For example, mid‐life vascular risk factors may increase the risk of late‐life brain atrophy and clinical Alzheimer’s disease (AD) by causing cerebrovascular disease, AD, or a combination of the two. Clarifying the relationship between mid‐life vascular risk and late‐life neuropathology is important for elucidating the mechanisms leading to both AD and vascular cognitive impairment.

In this study, we examined the association between mid‐ and late‐life vascular risk factor burden and neuropathology in old age in a community‐based sample. Both vascular and AD neuropathology were examined as outcomes.

## Methods

### Study design

**Figure 1:**
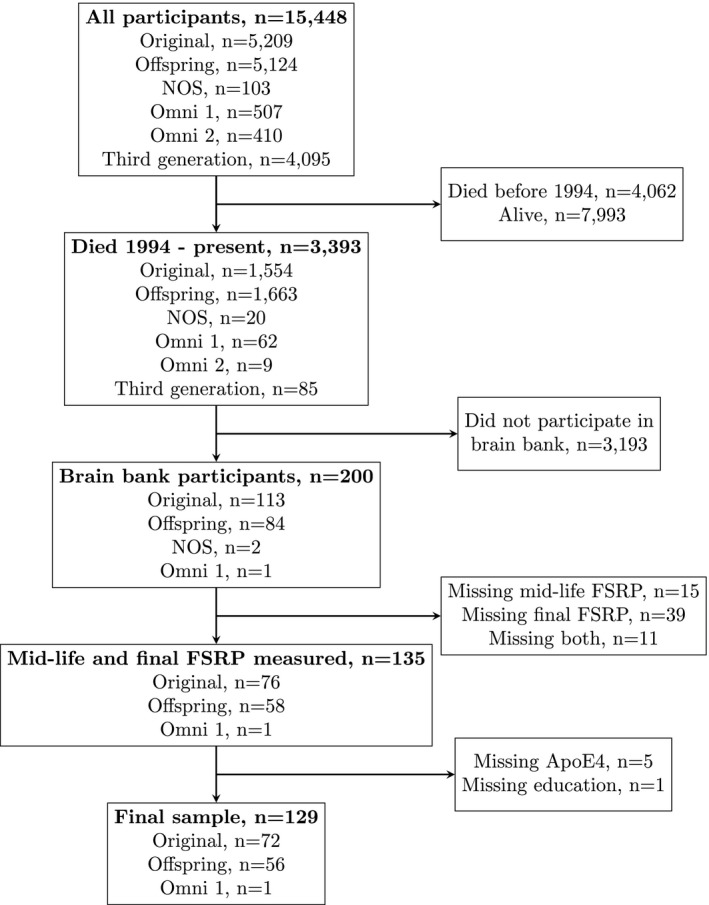
Flow diagram of study participants. NOS = New Offspring/Spouse cohort, FSRP = Framingham Stroke Risk Profile.

The FHS is a community‐based, longitudinal cohort study that began in 1948 in Framingham, Massachusetts.^4^, ^5^, ^6^ Additional details are provided in the Appendix S1 and Figure S1. The voluntary FHS brain donation program began in 1994.^7^ The present study includes participants from the Original, Offspring, and Omni 1 cohorts who have died, donated their brain to the FHS brain bank, had vascular risk factors measured both in mid‐life and within 10 years prior to death, and have information on education and presence of the Apolipoprotein E (ApoE) ɛ4 allele (Fig. 1).

All FHS participants provided written informed consent, and the study was approved by the Boston University Medical Center Institutional Review Board. Consent for autopsy at time of death was obtained from the next of kin.

### Vascular risk burden

Vascular risk burden was calculated using the revised Framingham Stroke Risk Profile (FSRP).^8^, ^9^, ^10^, ^11^, ^12^, ^13^, ^14^, ^15^ The revised FSRP score incorporates age, systolic blood pressure, current smoking status, antihypertensive medication use, prevalent diabetes mellitus, prevalent cardiovascular disease (CVD), and prevalent atrial fibrillation (AF), and is stratified by gender. We considered vascular risk burden at two separate time points: mid‐life and the closest examination before death. Mid‐life vascular risk burden was measured at the examination closest to and within 5 years of 55 years of age. Late‐life vascular risk burden was measured at the last attended exam, up to 10 years prior to death. Additional details are provided in the Appendix S1.

### Neuropathology outcomes

#### Neuropathological assessment

Brain autopsies were performed by board‐certified neuropathologists (ACM, VEA, TDS). Neuropathologists were blinded to any clinical or demographic information. Details of the neuropathological assessment have been discussed elsewhere.^7^ Tissue blocks were embedded in paraffin and evaluated with Luxol fast blue hematoxylin and eosin stains and immunohistochemistry in 24 brain regions, including frontal, temporal, parietal, and visual cortices; pre‐ and postcentral, dorsolateral middle frontal, and anterior and posterior cingulate gyri; olfactory bulb, caudate, putamen, nucleus accumbens, amygdala, entorhinal cortex, globus pallidus, substantia innominata, hippocampus at the level of the lateral geniculate, and thalamus.

#### Vascular lesions

Brains were assessed for vascular pathology, including the number of chronic infarcts, defined as macroscopic encephalomalacic lesions. We assessed cortical and subcortical regions separately. Due to the sparse distribution of multiple infarcts, we dichotomized cortical and subcortical infarcts as present versus absent.

Brains were also assessed semi‐quantitatively for the degree of cerebral arteriosclerosis in four regions of deep white matter and basal ganglia, atherosclerosis in the circle of Willis, and cerebral amyloid angiopathy (CAA) in the middle frontal, inferior parietal, superior temporal, and calcarine cortices. Semi‐quantitative scores for arteriosclerosis, atherosclerosis, and CAA ranged from none (0) to severe (3). A global CAA severity score was determined using NIA‐AA guidelines.^16^ Briefly, the absence of CAA was scored as 0, mild CAA (1) was defined as scattered Aβ‐positive vessels; moderate CAA (2) was defined as intense Aβ‐positivity; severe CAA (3) was defined as widespread intense Aβ‐positivity in vessels in multiple brain regions.

#### Neuritic plaque score

The density of neuritic plaques was determined within the dorsolateral frontal cortex, superior temporal gyrus, inferior parietal lobule, and calcarine cortex with Aß immunostaining (4G8; BioLegend, San Diego, CA; 1:100,000) and Bielschowsky silver stain.^7^ The overall density was used to determine the Consortium to Establish a Registry for Alzheimer's Disease (CERAD) neuritic plaque score, ranging from none (0) to frequent (3).^17^

#### Neurofibrillary tangles

The density of neurofibrillary tangles (NFTs) was determined with immunostaining for phosphorylated PHF‐tau (AT8; Pierce Endogen, Rockford, IL; 1:2000) as well as Bielschowsky silver staining. Tau pathology burden was assessed using the Braak stage for NFT pathology, which consisted of seven stages (Stage 0 – Stage VI), where a higher stage indicates greater disease extent and severity.^7^, ^18^ One participant without NFT (Stage 0, *n* = 1) was classified as Stage I in our analyses.

### Statistical analysis

We fit multivariable logistic models for dichotomous outcomes (presence of cortical infarcts and presence of subcortical infarcts upon autopsy) and multivariable proportional‐odds logistic models for ordered categorical outcomes (atherosclerosis, arteriosclerosis, Braak NFT stage, CAA, and CERAD neuritic plaque score upon autopsy). We assessed the proportional odds assumption via score tests comparing the likelihoods of proportional‐odds and multinomial models. The proportional odds assumption was met for all models.

We fit models for mid‐life vascular risk burden with and without adjustment for late‐life vascular risk burden. Models were adjusted for gender, presence of an ApoE ɛ4 allele, and elapsed time between mid‐life FSRP score and death. In secondary analyses, we fit models for final vascular risk burden before death instead of mid‐life to examine the association between late‐life vascular risk burden and each neuropathological outcome. These models were adjusted for gender, presence of an ApoE ɛ4 allele, and elapsed time between final FSRP score and death. We assessed potential interaction between vascular risk burden and gender by including multiplicative interaction terms in the models. In the presence of significant interaction, we also present odds ratios stratified by gender.

To address potential selection bias, we performed a sensitivity analysis in which we fit all models using inverse probability weighting (IPW).^19^, ^20^, ^21^ Participants were weighted by their inverse probability of participating in the brain bank program, given they were eligible to participate (died after the start of the FHS brain donation program in 1994). Further details are provided in the Appendix S1. We adjusted IPW models for presence of ApoE ɛ4 and elapsed time between FSRP score and death. We estimated variances using Taylor series variance estimation to account for the weights (SAS PROC SURVEYLOGISTIC). In additional sensitivity analyses, we adjusted for FHS cohort (Original vs. Offspring or Omni 1).

Analyses were performed using SAS version 9.4 (SAS Institute Inc., Cary, North Carolina) and R version 3.4 (R Foundation for Statistical Computing, Vienna, Austria). We considered results with p‐value < 0.05 statistically significant.

## Results

### Participant characteristics

The analysis sample consisted of 60 men and 69 women. The mean FSRP was lower and less variable at mid‐life (mean: 1.3, standard deviation: 0.8) than final measurement (mean: 20.3, standard deviation: 14.1). The median time interval between FSRP and death was 33.4 years at mid‐life and 4.4 years at final measurement before death. The overall sample characteristics are provided in Table 1, whereas the distributions of the outcome are available in Table 2.

**Table 1:** Characteristics of study sample.

Variable	Final sample (*n* = 129)
Demographic characteristics	
Women, *n* (%)	69 (53.5%)
Age at mid‐life measurement, median [Q1, Q3]	55.1 [54.2, 55.8]
Age at death, median [Q1, Q3]	84.1 [77.2, 87.2]
Education	
No high school degree, *n* (%)	14 (10.9%)
High school degree, *n* (%)	35 (27.1%)
Some college, *n* (%)	34 (26.4%)
College graduate, *n* (%)	46 (35.7%)
Clinical characteristics	
Mid‐life FSRP, mean ± SD	1.3 ± 0.8
Late‐life FSRP, mean ± SD	20.3 ± 14.1
Time (years) from mid‐life FSRP to death, median [Q1, Q3]	33.4 [26.3, 37.2]
Time (years) from final FSRP to death, median [Q1, Q3]	4.4 [3.0, 6.8]
ApoE ɛ4 carriers, *n* (%)	32 (24.8%)
Clinical dementia rating	
None (0.0), *n* (%)	48 (37.8%)
Very mild (0.5), *n* (%)	22 (17.3%)
Mild (1.0), *n* (%)	12 (9.5%)
Moderate (2.0), *n* (%)	16 (12.6%)
Severe (3.0), *n* (%)	29 (22.8%)
FSRP components at mid‐life	
Systolic blood pressure, mean ± SD	124 ± 14
Current smoking, *n* (%)	32 (24.8%)
Antihypertensive medication, *n* (%)	10 (7.8%)
Prevalent diabetes mellitus, *n* (%)	2 (1.6%)
Prevalent cardiovascular disease, *n* (%)	3 (2.3%)
Prevalent atrial fibrillation, *n* (%)	3 (2.3%)
FSRP components at final measurement	
Systolic blood pressure, mean ± SD	136 ± 20
Current smoking, *n* (%)	6 (4.7%)
Antihypertensive medication, *n* (%)	69 (53.5%)
Prevalent diabetes mellitus, *n* (%)	13 (10.1%)
Prevalent cardiovascular disease, *n* (%)	32 (25.0%)
Prevalent atrial fibrillation, *n* (%)	24 (18.6%)

**Table 2:** Distribution of neuropathological outcomes.

Neuropathological outcome	Final sample (*n* = 129)
Cerebrovascular pathology	
Present cortical infarcts, n (%)	12 (9.3%)
Present subcortical infarcts, n (%)	25 (19.4%)
Atherosclerosis	
None, *n* (%)	29 (22.5%)
Mild, *n* (%)	67 (51.9%)
Moderate, *n* (%)	24 (18.6%)
Severe, *n* (%)	9 (7.0%)
Arteriosclerosis	
None, *n* (%)	36 (28.1%)
Mild, *n* (%)	47 (36.7%)
Moderate, *n* (%)	33 (25.8%)
Severe, *n* (%)	12 (9.4%)
Proteinopathy	
Braak stage	
Stage 0–I, *n* (%)	29 (22.5%)
Stage II, *n* (%)	32 (24.8%)
Stage III, *n* (%)	26 (20.2%)
Stage IV, *n* (%)	12 (9.3%)
Stage V, *n* (%)	15 (11.6%)
Stage VI, *n* (%)	15 (11.6%)
Cerebral amyloid angiopathy	
None, *n* (%)	29 (22.5%)
Mild, *n* (%)	67 (51.9%)
Moderate, *n* (%)	24 (18.6%)
Severe, *n* (%)	9 (7.0%)
CERAD neuritic plaque score	
None, *n* (%)	34 (26.4%)
Sparse, *n* (%)	37 (28.7%)
Moderate, *n* (%)	27 (20.9%)
Frequent, *n* (%)	31 (24.0%)

### Associations between mid‐life vascular risk burden and neuropathology

All results for associations between mid‐life vascular risk factor burden and neuropathology are available in Table 3. Mid‐life vascular risk burden was significantly associated with cortical infarcts, subcortical infarcts, greater atherosclerosis score, and greater arteriosclerosis score. For each one‐point increase in vascular risk burden, the odds of cortical infarcts increased 3‐fold (OR [95% CI]: 3.99 [1.65, 9.65]), whereas the odds of subcortical infarcts, greater atherosclerosis score, and greater arteriosclerosis score almost doubled (OR [95% CI]: 1.95 [1.07, 3.54], 1.88 [1.17, 3.00], and 1.74, [1.09, 2.79] respectively). Associations remained significant and similar in magnitude after additional adjustment for late‐life vascular risk burden. Mid‐life vascular risk burden was not significantly associated with any proteinopathy outcomes (Braak NFT stage, CAA, CERAD plaque score). In the majority of models, a linear association between time from vascular risk to death and the log odds of the outcome was appropriate. Models for mid‐life vascular risk with atherosclerosis and arteriosclerosis included quadratic terms. We did not identify any significant interactions between midlife vascular risk burden and gender.

**Table 3:** Associations of vascular risk burden and neuropathology outcomes.

Outcome	Mid‐life vascular risk Model 1		Mid‐life vascular risk Model 2		Late‐life vascular risk Model 3	
	OR (95% CI)	*P*	OR (95% CI)	*P*	OR (95% CI)	*P*
Cerebrovascular pathology						
Cortical infarcts	3.99 (1.65, 9.65)	0.002*	3.83 (1.55, 9.45)	0.004*	1.04 (1.00, 1.08)	0.049*
Subcortical infarcts	1.95 (1.07, 3.54)	0.029*	1.85 (1.01, 3.40)	0.047*	1.02 (0.99, 1.06)	0.129
Atherosclerosis	1.89 (1.17, 3.06)	0.009*	1.90 (1.16, 3.10)	0.011*	1.02 (1.00, 1.05)	0.070
Arteriosclerosis	1.86 (1.15, 3.02)	0.012*	1.78 (1.09, 2.90)	0.022*	1.03 (1.00, 1.05)	0.024*
Proteinopathy						
Braak NFT stage	1.15 (0.73, 1.82)	0.554	1.18 (0.74, 1.89)	0.484	1.03 (1.01, 1.05)	0.012*
Cerebral amyloid angiopathy	1.15 (0.71, 1.86)	0.575	1.19 (0.72, 1.95)	0.494	1.02 (1.00, 1.05)	0.061
CERAD neuritic plaque score	0.86 (0.54, 1.37)	0.525	0.86 (0.54, 1.39)	0.547	1.02 (1.00, 1.05)	0.052

OR, Odds ratio; CI, confidence interval; *P*, *P*‐value; CERAD, Consortium to Establish a Registry for Alzheimer's Disease.

Model 1: Adjusted for gender, presence of ApoE ɛ4, and elapsed time between mid‐life vascular risk and death (with quadratic term for atherosclerosis and arteriosclerosis).

Model 2: Model 1 additionally adjusted for late‐life vascular risk.

Model 3: Adjusted for gender, presence of ApoE ɛ4, and elapsed time between late‐life vascular risk and death.

* *P* < 0.05.

### Associations between late‐life vascular risk burden and neuropathology

Associations between late‐life vascular risk factor burden and brain pathology were much smaller in magnitude as compared to mid‐life vascular risk burden scores and brain pathology (Table 3). For each one‐point increase in late‐life vascular risk burden, the odds of cortical infarcts increased by 4% (95% CI: 1.00, 1.08) and the odds of greater arteriosclerosis score increased by 3% (95% CI: 1.00, 1.05). Higher vascular risk factor burden in late‐life was also associated with higher Braak stage; the odds of higher Braak stage increased by 3% for each one‐point increase in late‐life vascular risk burden (95% CI: 1.01, 1.05). All models included time from late‐life vascular risk to death as a continuous covariate.

**Figure 2:**
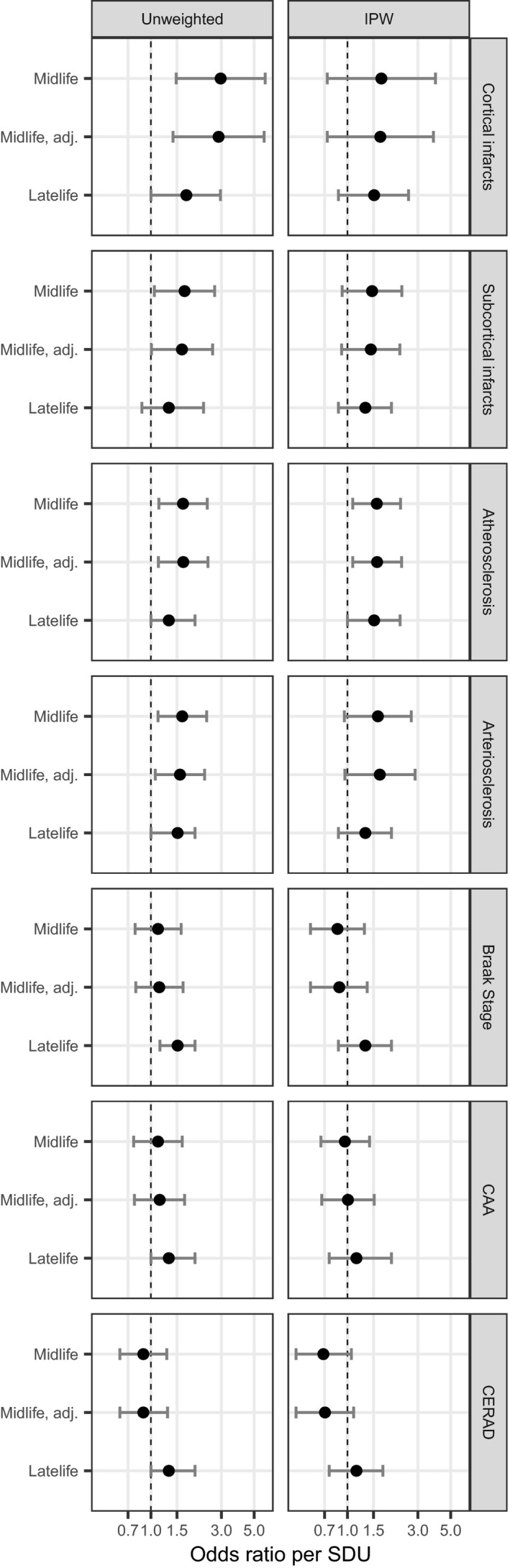
Adjusted odds ratios for associations between mid‐life and late‐life vascular risk and neuropathologic outcomes. All unweighted models are adjusted for gender, presence of ApoE ɛ4, and elapsed time between vascular risk and death. IPW models are adjusted for presence of ApoE ɛ4 and elapsed time between vascular risk and death. IPW = inverse probability weighting, CAA = cerebral amyloid angiopathy, CERAD = Consortium to Establish a Registry for Alzheimer's Disease neuritic plaque score, mid‐life = vascular risk factors measured in mid‐life, mid‐life adj. = vascular risk factors measured in mid‐life with adjustment for late life vascular risk factors, late‐life = vascular risk factors measured in late life. SDU = odds ratio per standard deviation unit difference in the Framingham Stroke Risk Profile score.

In Figure 2, we plot the adjusted odds ratios per standard deviation unit (SDU) increase in vascular risk burden (SDU: 0.8 for midlife vascular risk and 14.1 for late‐life vascular risk) to facilitate comparisons of mid‐life and late‐life vascular risk associations with neuropathology. For cortical infarcts, subcortical infarcts, and atherosclerosis, the magnitude of association per SDU increase in mid‐life vascular risk was larger than the magnitude of association per SDU increase in late‐life vascular risk. For proteinopathy outcomes, the magnitude associations per SDU increase in late‐life vascular risk were larger than those for mid‐life vascular risk.

We identified significant interactions between late‐life vascular risk burden and gender for cortical infarcts, arteriosclerosis, and CAA (*P*‐value = 0.011, 0.001, and 0.002 respectively). In stratified analyses, ORs were significant in men (OR [95% CI]: 1.24 [1.06, 1.45], 1.09 [1.05, 1.14], and 1.08 [1.04, 1.13], respectively) but not in women (OR [95% CI]: 0.99 [0.93, 1.06], 1.00 [0.97, 1.03], and 0.99 [0.96, 1.02] respectively).

### Sensitivity analyses using inverse probability weighting

We fit a logistic model to obtain inverse probability weights for our sensitivity analyses. Year of birth and education were significantly associated with the odds of brain bank participation (*P* = 0.009 and *P* < 0.001, respectively), while gender and cohort membership were not. The odds of brain bank participation decreased by 3% for each year increase in year of birth. Compared to participants who did not graduate high school, the odds of brain bank participation were 85% greater in high school graduates, 195% greater in participants with some college, and 441% greater in college graduates.

IPW analyses of mid‐life vascular risk burden with cerebrovascular pathology had similar magnitude, but only the association with atherosclerosis was statistically significant (Table 4). Similarly, results for late‐life vascular risk were generally consistent between unweighted and IPW analyses (Fig. 2). In additional sensitivity analyses adjusting for FHS cohort, results were similar to the original findings (Table S1).

**Table 4:** Sensitivity analysis: associations of vascular risk burden and neuropathology outcomes using inverse probability weighting.

Outcome	Mid‐life vascular risk Model 1		Mid‐life vascular risk Model 2		Late‐life vascular risk Model 3	
	OR (95% CI)	*P*	OR (95% CI)	*P*	OR (95% CI)	*P*
*Cerebrovascular pathology*						
Cortical infarcts	1.96 (0.67, 5.74)	0.100	1.92 (0.67, 5.51)	0.228	1.03 (0.99, 1.07)	0.115
Subcortical infarcts	1.63 (0.90, 2.94)	0.142	1.59 (0.89, 2.83)	0.115	1.02 (0.99, 1.05)	0.210
Atherosclerosis	1.79 (1.11, 2.87)	0.017*	1.80 (1.11, 2.93)	0.019*	1.03 (1.00, 1.06)	0.045*
Arteriosclerosis	1.83 (0.94, 3.55)	0.074	1.90 (0.95, 3.83)	0.071	1.02 (0.99, 1.05)	0.224
*Proteinopathy*						
Braak NFT stage	0.82 (0.48, 1.40)	0.466	0.85 (0.48, 1.48)	0.562	1.02 (0.99, 1.05)	0.206
Cerebral amyloid angiopathy	0.95 (0.59, 1.55)	0.852	1.01 (0.60, 1.70)	0.974	1.01 (0.98, 1.05)	0.412
CERAD neuritic plaque score	0.62 (0.36, 1.08)	0.090	0.64 (0.36, 1.13)	0.126	1.01 (0.98, 1.04)	0.600

OR, Odds ratio, CI, confidence interval; *P*: *P*‐value; CERAD, Consortium to Establish a Registry for Alzheimer's Disease.

Model 1: Adjusted for presence of ApoE ɛ4 and elapsed time between mid‐life vascular risk and death (with quadratic term for atherosclerosis and arteriosclerosis).

Model 2: Model 1 additionally adjusted for late‐life vascular risk.

Model 3: Adjusted for presence of ApoE ɛ4, and elapsed time between late‐life vascular risk and death.

* *P* < 0.05.

## Discussion

We examined the relationship of mid‐ and late‐life vascular risk factor burden with neuropathology in old age in a well‐characterized community‐based cohort. Mid‐life vascular risk factor burden was associated with cerebrovascular neuropathology at autopsy a median of 33.4 years later, even after adjustment for vascular risk burden proximal to death. Mid‐life vascular risk was not associated with AD neuropathology, although higher late‐life vascular risk was associated with a greater burden of NFT. Therefore, in our cohort, mid‐life vascular risk factors independently predicted cerebrovascular but not AD neuropathology.

A number of studies have examined the relationship between vascular risk factors and neuropathology. However, such studies are limited to measurement of vascular risk factors in old age. In contrast, few studies have performed detailed neuropathological examination of a community‐based cohort with assessments of vascular risk factors extending back to midlife. Our findings are novel because of the long follow‐up, which allows us to assess vascular burden at multiple points in life, including the midlife and late life as studied here. One of our main findings was that vascular risk factor burden more strongly associated with cerebrovascular pathology when vascular risk factors were measured in mid as compared to later life. It is plausible that a higher vascular risk burden in mid‐life reflects an early vascular aging, whereas a higher vascular risk burden before death is consistent with the typical patterns of vascular aging observed in Western populations.^1^, ^22^ In support of this notion, the FSRP had a small range at mid‐life but a relatively larger range in later life (Table 1). Living with a high vascular risk factor burden from a young age also provides a longer exposure window by which vascular risk factors may cause the insidious accumulation of cerebrovascular disease. Additionally, the distributions of vascular risk factors differed between mid‐ and late‐life measurements. In our study, the prevalence of antihypertensive medication, diabetes, CVD, and AF, increased from mid‐life to final measurement, while the prevalence of smoking decreased from mid‐life to final measurement.

Mid‐life vascular risk factors have been associated with late‐life cognitive decline and a higher risk of clinical AD.^11^, ^23^, ^24^, ^25^, ^26^ However, the underlying mechanisms are not entirely clear. Vascular risk factors are well known to increase risk for silent cerebrovascular disease and stroke,^11^, ^23^ both of which are powerful risk factors for dementia, including clinical AD.^24^, ^25^, ^26^ However, it is less clear whether mid‐life vascular risk factors are directly associated with AD pathology. In our cohort, mid‐life vascular risk factor burden was not related to proteinopathy outcomes. The lack of these associations cannot be attributed solely to a lack of statistical power, since the direction of the odds ratio for neuritic plaque score indicated an inverse association. Many of the IPW odds ratios also indicated inverse associations of vascular risk with proteinopathy outcomes. Consistent with our findings, data from the Statistical Modeling of Aging and Risk of Transition (SMART) consortium demonstrated an association between diabetes status and cerebrovascular pathology, but not AD pathology.^27^ Other studies have observed less neuritic plaques and NFT in persons with diabetes as compared to persons without diabetes.^28^, ^29^, ^30^ In contrast, recent data from the Atherosclerosis Risk in the Communities (ARIC) study found that an increasing number of vascular risk factors in mid‐, but not late‐life, associated with a higher amyloid burden on PET imaging more than 20‐years later.^31^ Although suggestive of an association between higher vascular risk and greater neuritic plaque density, amyloid PET tracer retention can also indicate amyloid from other sources, such as vascular amyloid in CAA.^32^ Overall, data on the relationship between mid‐life vascular risk and later life AD is scarce and in need of further study.

Although mid‐life vascular risk did not relate to AD pathology in our cohort, higher vascular risk in late‐life did nominally associate with a greater burden of NFT. Although not significant, effect sizes also pointed towards a positive relationship between vascular risk factor burden in old age and brain amyloid accumulation. Consistent with this, others have shown that the Framingham Coronary Heart Disease Risk Profile score is positively associated with amyloid burden on PET imaging in a small, elderly non‐demented sample (mean age of 79 years).^33^ Other data suggests that vascular risk factor burden and AD may relate to cognitive function through distinct pathways.^34^ For example, data from the National Alzheimer's Coordinating Center indicates that the association between the FSRP and cognitive function was strongest in persons who were not positive for AD neuropathology at autopsy.^34^

Taken together with our data, the above findings suggest that mid‐life vascular risk factors may influence the risk of clinical dementia by causing the accumulation of cerebrovascular rather than Alzheimer’s pathology. However, one cannot ignore the fact that vascular and neurodegenerative pathologies frequently co‐occur in patients with dementia.^35^ Moreover, the link between late‐life vascular risk factor burden and NFT deposition suggests an age‐dependent interaction between vascular risk and AD pathology. These relationships require further study across the lifespan.

There are several limitations to our study. First, while our sample size may not be considered small for neuropathology studies, we only observed a small number of cortical and subcortical infarcts (Table 2). Thus, some of our analyses may have been underpowered. Second, our sample was comprised mostly of participants of European descent, limiting the generalizability of our results to other ethnic populations. Third, brain bank studies are subject to potential selection bias since only a small percentage of participants consent to brain donation. Although we employed IPW in a sensitivity analysis to confirm the generalizability of our sample to the greater FHS sample, our model may not have accounted for all possible factors related to brain donation. The results of IPW analyses were slightly smaller in magnitude than the results of unweighted analyses but were consistent overall, which strengthens the validity of our findings. Finally, it is possible that higher vascular risk burden increased the risk of mortality earlier in life. As the prevalence of AD pathology increase with age, premature death due to vascular disease may have lessened our ability to observe associations between midlife vascular risk factor burden and late‐life AD pathology. In other words, persons with low risk of death due to cardiovascular disease may be the most likely to survive into old age, when AD dementia is most prevalent. This may explain why we sometimes observed inverse associations between vascular risk burden and AD pathology.

The FSRP is a widely validated predictor of clinical stroke.^11^ Our data add support to the utility of the FSRP as a predictor of other cerebrovascular pathologies, including atherosclerosis and arteriosclerosis. Our data also support the notion of minimizing vascular risk factors from an early age to limit the accumulation of cerebrovascular disease and the resultant cognitive sequelae. However, it is still unclear as to whether mid‐life vascular risk directly impacts the development or progression of AD pathology.

## Author Contributions

SCC, MRR, AB, and SS designed and conceptualized the study. SCC and JJH performed the statistical analyses. SCC and MPP drafted the manuscript. ACM, VEA, and TDS performed brain autopsies and assessment of outcomes. ACM, VEA, TDS, JJH, AB, and SS played major roles in the acquisition of data. All authors revised the manuscript.

## Conflicts of Interest

Ms. Conner reports no disclosures. Dr. Pase reports no disclosures. Dr. Carneiro reports no disclosures. Dr. Raman reports no disclosures. Dr. McKee reports no disclosures. Dr. Alvarez reports no disclosures. Dr. Walker reports no disclosures. Dr. Satizabal reports no disclosures. Dr. Himali reports no disclosures. Dr. Stein reports no disclosures. Dr. Beiser reports no disclosures. Dr. Seshadri reports no disclosures.

## Supporting information


**Appendix S1.** Methods section, continued and results for a sensitivity analysis adjusting for cohort membership.

**Figure S1.** Causal diagram of brain bank selection process for inverse probability weight model.
**Table S1.** Sensitivity analysis: associations of vascular risk burden and neuropathology outcomes, adjusted for cohort.
