# Supplementary material for: Mid‐life and late‐life vascular risk factor burden and neuropathology in old age
Source: Ann Clin Transl Neurol. 2019 Nov 5;6(12):2403–12. doi: 10.1002/acn3.50936 (PMC6917310; doi:10.1002/acn3.50936)
Supplement: Supplementary file 1 — Appendix S1. Methods section, continued and results for a sensitivity analysis adjusting for cohort membership. [file ACN3-6-2403-s001.docx]

**Appendix**

**Mid-life and late-life vascular risk factor burden and neuropathology in old age**

Sarah C. Conner MPH^1,2^, Matthew P. Pase PhD^1,3,4,5^, Herman Carneiro MD MSc^1,6^, Mekala R. Raman PhD^7^, Ann C. McKee MD^7,8,9,10,11^, Victor E. Alvarez MD^8,9,10,11^, Jamie M. Walker^12^, Claudia L. Satizabal PhD^1,7,12^, Jayandra J. Himali PhD^1,2,7^, Thor D. Stein MD, PhD^8,9,10,11^, Alexa Beiser PhD^1,2,7^, Sudha Seshadri MD^1,7,12^

**Figures**

Figure A1 Title: Causal diagram of brain bank selection process for inverse probability weight model.

**Methods, continued.**

**Study design and participants**

The FHS began in 1948 with 5,209 participants (Original cohort). In 1971, children of the Original cohort and their spouses were recruited (Offspring cohort), and two ethnically diverse cohorts were recruited in 1994 (Omni 1) and 2003 (Omni 2). In 2002, grandchildren of the Original cohort and children of the Offspring cohort were recruited (Third Generation). Participants attend clinical examinations approximately every two years (Original cohort) or four to six years (all other cohorts).^5, 6^

**Vascular risk burden**

Vascular risk burden was calculated using the revised Framingham Stroke Risk Profile (FSRP).^11^ The original FSRP was developed in 1991 in participants of the FHS to estimate stroke risk and identify vascular risk factors, which has been widely used by clinicians to assess vascular risk burden.^12-16^ In 2017, the FSRP was updated to reflect more contemporary associations between risk factors and stroke incidence as well as an overall decline in the age-adjusted incidence of stroke, and was validated in two other large cohorts.^8-15^ The revised FSRP score incorporates age, systolic blood pressure, current smoking status, antihypertensive medication use, prevalent diabetes mellitus, prevalent cardiovascular disease (CVD), and prevalent atrial fibrillation (AF), and is stratified by sex.

*Vascular risk burden at mid-life and before death*

We considered vascular risk burden at two separate time points: mid-life and the closest examination before death. Mid-life vascular risk burden was measured at the examination closest to and within 5 years of 55 years of age. Late-life vascular risk burden was measured at the last attended exam, up to 10 years prior to death.

SBP is the mean of two physician measurements on the participant’s left arm. Current smoking status was defined as smoking in the year prior to examination. Prevalent diabetes was defined as use of insulin or oral hypoglycemic medication before or at the examination, fasting plasma blood glucose greater than or equal to 7 mmol/l, or casual plasma blood glucose greater than or equal to 11.1 mmol/l. Prevalent CVD was defined as coronary artery disease, congestive heart failure, or peripheral vascular disease documented before or at the examination, while prevalent AF was defined as atrial fibrillation or flutter determined by a cardiologist via electrocardiography before or at examination.

**Statistical analysis**

We examined the functional form of the association between vascular risk burden and time from FSRP to death and the log odds of the outcome by plotting the beta coefficients for binned time (four categories). If the association between binned time and log odds of the outcome appeared quadratic, we included a quadratic term. We confirmed our model choice with the deviance goodness-of-fit test, where a p-value≥0.05 indicates adequate model fit.

To address potential selection bias, we performed a sensitivity analysis in which we fit all models using inverse probability weighting (IPW).^19^ Participants were weighted by their inverse probability of participating in the brain bank program, given they were eligible to participate (died after the start of the FHS brain donation program in 1994). Predicted probabilities were obtained using logistic models including year of birth, sex, education, and an indicator for FHS Original cohort membership (due to low frequencies of brain bank participants in certain cohorts). Predictors of brain bank participation were selected based on previous literature.^19^ A diagram of the associations and selection process is available in the supplementary material (Figure A1). Weights were stabilized (by multiplying by the probability of selection status) to avoid any extremely large weights.^20, 21^ We adjusted IPW models for presence of ApoE ɛ4 and elapsed time between FSRP score and death. We estimated variances using Taylor series variance estimation to account for the weights (SAS PROC SURVEYLOGISTIC). In additional sensitivity analyses, we adjusted for FHS cohort (Original vs. Offspring or Omni 1).

| **Table A1. Sensitivity analysis: associations of vascular risk burden and neuropathology outcomes, adjusted for cohort.** | | | | | | |
| --- | --- | --- | --- | --- | --- | --- |
|  | **Mid-life vascular risk**  **Model 1** | | **Mid-life vascular risk**  **Model 2** | | **Late-life vascular risk**  **Model 3** | |
| **Outcome** | **OR (95% CI)** | **p** | **OR (95% CI)** | **p** | **OR (95% CI)** | **p** |
| ***Cerebrovascular pathology*** |  |  |  |  |  |  |
| Cortical infarcts | 3.97 (1.64, 9.63) | 0.002^*^ | 3.79 (1.53, 9.42) | 0.004^*^ | 1.04 (1.00, 1.09) | 0.066 |
| Subcortical infarcts | 2.18 (1.17, 4.09) | 0.015^*^ | 2.11 (1.11, 4.02) | 0.022^*^ | 1.01 (0.98, 1.05) | 0.572 |
| Atherosclerosis | 1.88 (1.16, 2.99) | 0.010^*^ | 1.85 (1.13, 3.04) | 0.014^*^ | 1.03 (1.00, 1.06) | 0.027^*^ |
| Arteriosclerosis | 1.85 (1.14, 3.05) | 0.013^*^ | 1.75 (1.07, 2.87) | 0.027^*^ | 1.04 (1.01, 1.06) | 0.012^*^ |
| ***Proteinopathy*** |  |  |  |  |  |  |
| Braak NFT stage | 1.14 (0.71, 1.81) | 0.591 | 1.15 (0.72, 1.85) | 0.559 | 1.03 (1.00, 1.06) | 0.027^*^ |
| Cerebral amyloid angiopathy | 1.15 (0.71, 1.86) | 0.578 | 1.19 (0.72, 1.95) | 0.495 | 1.01 (0.99, 1.04) | 0.364 |
| CERAD neuritic plaque score | 0.88 (0.55, 1.40) | 0.581 | 0.89 (0.55, 1.43) | 0.621 | 1.01 (0.98, 1.04) | 0.439 |

OR: Odds ratio, CI: confidence interval, P: p-value, CERAD: Consortium to Establish a Registry for Alzheimer's Disease.

^*^p<0.05.

Model 1: Adjusted for sex, presence of ApoE ɛ4, elapsed time between mid-life vascular risk and death (with quadratic term for atherosclerosis and arteriosclerosis), and cohort (Generation 1 vs. Offspring or Omni 1).

Model 2: Model 1 additionally adjusted for late-life vascular risk.

Model 3: Adjusted for presence of ApoE ɛ4, and elapsed time between late-life vascular risk and death
