# Supplementary material for: Mid‐life and late‐life vascular risk factor burden and neuropathology in old age
Source: Ann Clin Transl Neurol. 2019 Nov 5;6(12):2403–12. doi: 10.1002/acn3.50936 (PMC6917310; doi:10.1002/acn3.50936)
Supplement: Supplementary file 2 — Figure S1. Causal diagram of brain bank selection process for inverse probability weight model. Table S1. Sensitivity analysis: associations of vascular risk burden and neuropathology outcomes, adjusted for cohort. [file ACN3-6-2403-s002.pdf]

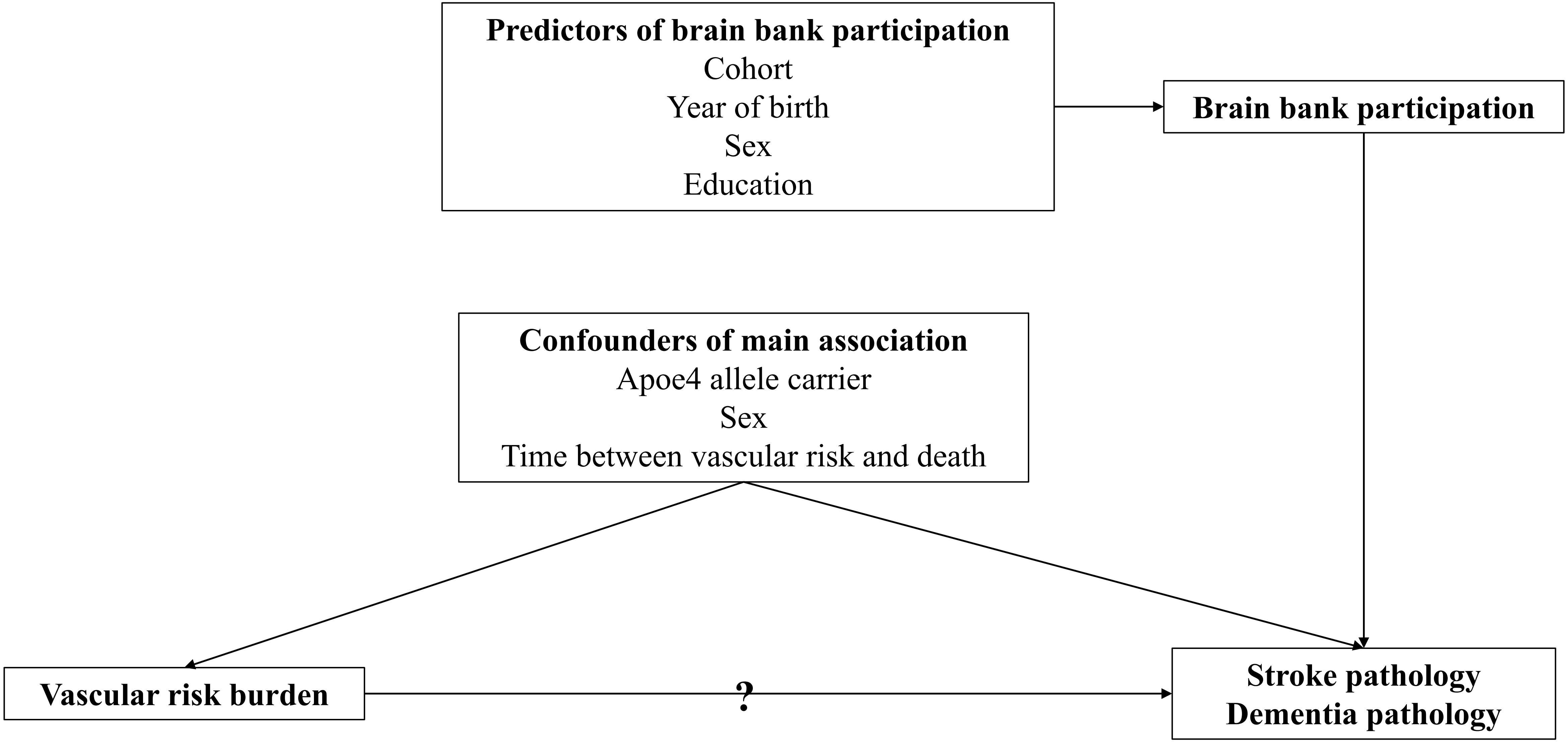
Predictors of brain bank participation
Cohort
Year of birth
Sex
Education
Brain bank participation
Confounders of main association
Apoe4 allele carrier
Sex
Time between vascular risk and death
Vascular risk burden
?
Stroke pathology
Dementia pathology
